# Trends and factors associated with declining lifetime fertility among married women in Kenya between 2003 and 2014: an analysis of Kenya demographic health surveys

**DOI:** 10.1186/s12889-023-15620-z

**Published:** 2023-04-20

**Authors:** James Orwa, Samwel Maina Gatimu, Paulino Ariho, Marleen Temmerman, Stanley Luchters

**Affiliations:** 1 Department of Public Health and Primary Care, Faculty of Medicine and Health Sciences, Ghent University, Ghent, Belgium; 2 Department of Population Health, Aga Khan University, Nairobi, Kenya; 3 Department of Population Health Sciences, Aga Khan University, P.O. Box 30270 – 00100, Nairobi, Kenya; 4 Diabetic Foot Foundation of Kenya, Nairobi, Kenya; 5 Department of Population Studies, School of Statistics and Planning, Makerere University, Kampala, Uganda; 6 Centre of Excellence for Women and Child Health, Aga Khan University, Nairobi, Kenya; 7 Centre for Sexual Health and HIV/AIDS Research (CeSHHAR), Harare, Zimbabwe; 8 Liverpool School of Tropical Medicine (LSTM), Liverpool, UK

**Keywords:** Cumulative fertility, Marital fertility, Kenya, Decomposition, Demographic transition, Repeat cross-sectional surveys

## Abstract

**Background:**

Globally, fertility has declined in the last three decades. In sub-Saharan Africa Including Kenya, this decline started more recent and at a slower pace compared to other regions. Despite a significant fertility decline in Kenya, there are disparities in intra- and interregional fertility. Reduction in lifetime fertility has health benefits for both the mother and child, thus it is important to improve women and children health outcomes associated with high fertility. The study, therefore evaluated the factors associate with change in lifetime fertility among married women of reproductive age in Kenya between 2003 and 2014.

**Methods:**

The study used the Kenya Demographic and Health Survey (KDHS) datasets of 2003, 2008 and 2014. Analysis of variance (ANOVA) was used to calculate the mean number of children ever born and to assess the change in fertility across different factors. Poisson regression model with robust standard errors was used to study the relationship between number of children ever born (lifetime fertility) and independent variables. A Poisson-based multivariate decomposition for the nonlinear response model was performed to identify and quantify the contribution of demographic, socioeconomic and reproductive correlates, to the change in lifetime fertility between 2003 and 2014.

**Results:**

The study included 3,917, 4,002, and 7,332 weighted samples of women of reproductive age in 2003, 2008, and 2014, respectively. The mean number of children born declined from 3.8 (95% CI: 3.6–3.9) in 2003 to 3.5 (95% CI: 3.4–-3.7) in 2008 and 3.4 (95% CI: 3.3–3.4) in 2014 (p = 0.001). The expected number of children reduced with the age at first sexual intercourse, the age at first marriage across the survey years, and household wealth index. Women who had lost one or more children in the past were likely to have increased number of children. The changes in the effects of women’s characteristics between the surveys explained 96.4% of the decline. The main contributors to the change in lifetime fertility was the different in women level of education.

**Conclusion:**

The lifetime fertility declined by one-tenth between 2003 and 2014; majorly as a result of the effects of characteristics of women in terms of level of education. These highlights a need to implement education policies that promotes women education focuses on gender equality and women empowerment. Continuous strengthening of the healthcare systems (access to quality antenatal care, skilled delivery, and postpartum care) to reduce child mortality is essential.

## Background

Fertility is one of the most important determinants of the size, structure, and composition of the human population [1]. Global fertility has been on the decline in Europe and North America since the 1900s, with a similar trend observed in Latin America and Asia over the past two decades [2]. In sub-Saharan Africa (SSA), fertility decline began much later, with stagnation experienced in the late 1990s and early 2000s [2]. Specifically, between 1950 and 2015, total fertility rates (TFR) in Asia and Latin America declined from 5.8 to 5.9 children per woman respectively to 2.2 children per woman. The African fertility during that period declined from 6.6 to 4.7 children per woman [3]. Schoumaker, et al. [4] observed a fertility stall in SSA that was first observed in Ghana and Kenya in the early 2000s, later in other sub-Saharan African countries [4]. A stall in fertility is defined as an ongoing fertility transition interrupted by a period of no significant change before a country reaches the end of the transition [5].

Conventional demographic transition theory, as developed by Thompson in 1929 and Notestein in 1945 [6], partially explains the decline in fertility in modern society. In the early stages of the demographic transition, high fertility resulted from the desire for large family sizes to provide family labour for farming and protection in old age, especially in SSA countries. In addition, high child mortality in the pre-modern era led couples to have many children to mitigate loss. The shift to low fertility in modern society is associated with urbanisation, industrialisation, and economic changes, resulting in fewer number of children. The infant mortality has declined as a result of improvement in the healthcare system (improved access to quality antenatal care, skilled birth deliveries, and postpartum care) [7]. In addition to these, through diffusion of new technologies, ideas and behaviour on birth control led to the fertility transition, as changes in reproductive behaviour proceeded much faster than the economic changes [6].

High fertility, defined as having five or more children, in sub-Saharan Africa is associated with low use of contraception, increased unmet need for family planning, an increase in preference for larger families, an increase in adolescent fertility [8, 9] and a disruption of female education [10]. On the other hand, previous studies have shown that socioeconomic factors such as urban residence, increase in education for both women and men, no history of infant mortality experience, and increase in age at first sexual intercourse, first birth or marriage have been associated with low fertility [11–13]. According to Bongaarts(1978), age at first marriage is considered an important proximate determinant of fertility [14]. Marriage age is not only been linked with the declined fertility in Europe and North America, but has been identified as one explanations to the high fertility levels observed In Africa and Asia where early marriages is still a common practice [15]. In the developing world, the main purpose of marriage is to have children which leads to early childbearing in women who marries at a younger ages [16, 17].

**Fig. 1:**
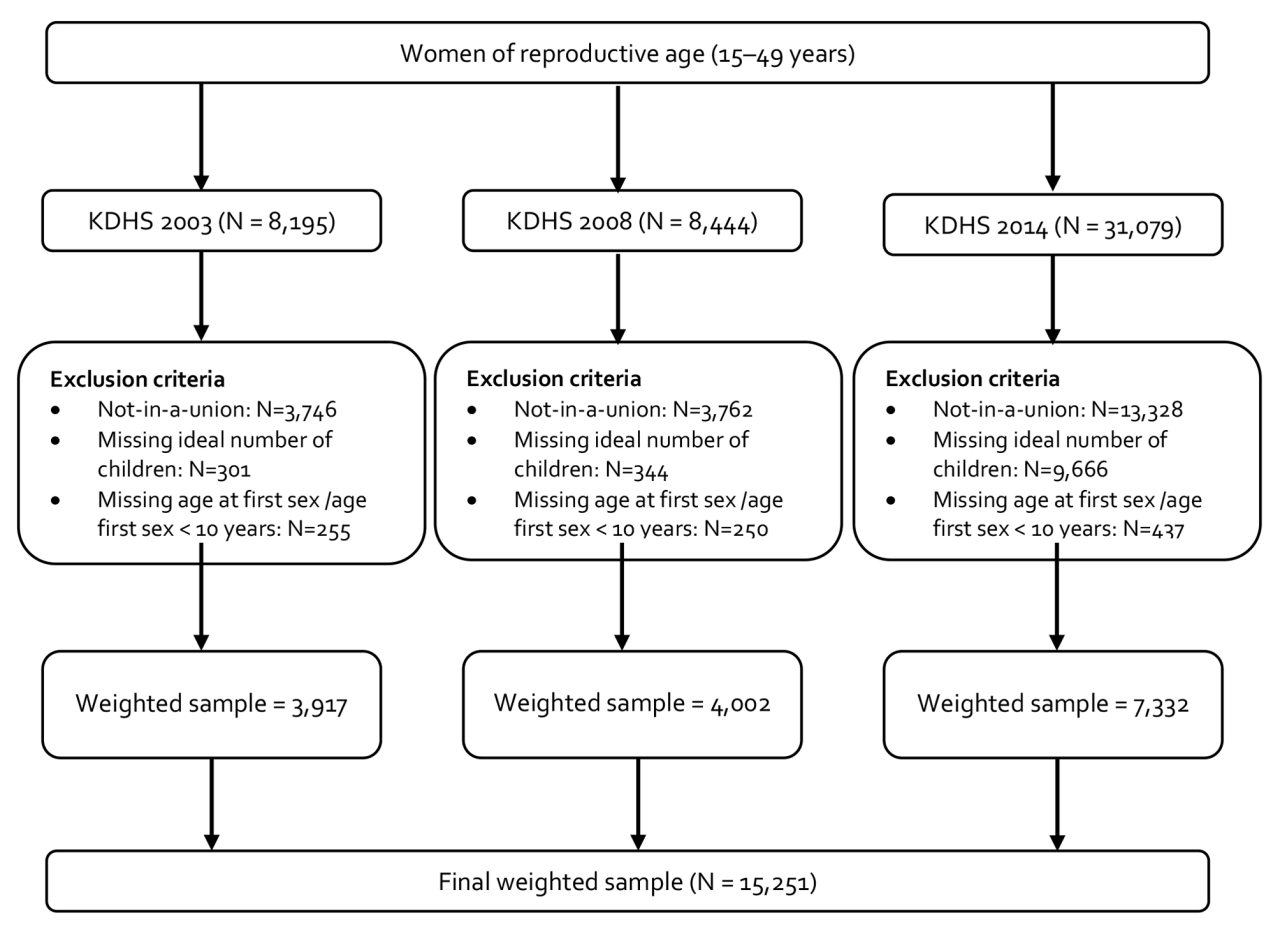
Flowchart highlighting the selection of the final sample size

High fertility poses a health risk for children and their mothers. Children from higher-order of births are likely to be at greater risk of dying during infancy and early childhood compared to children of lower orders. In resource limited settings where wealth is generated through wages, older children always compete for food with the younger siblings resulting into stunting growth among the young ones [18]. Maternal mortality is also more likely at higher pregnancy orders due to maternal depletion syndrome [19] which could lead to pregnancy loss or low birth weight babies. The syndrome may also trigger other maternal morbidities such as anemia, edema, and severe bleeding after childbirth and delivery complications [20, 21].

Kenya saw a decline in the total fertility rate among women of reproductive age from 4.9 to 2003 to 3.7 children per woman in 2014 [22]. The decline was preceded by a stagnant fertility rate of 4.7 and 4.6 children per woman in 1998 and 2009, respectively [22]. Similarly, the average number of children born remained relatively higher than the replacement level of 2.1 children at 3.8 in 2003 and 3.4 in 2014 among married women due to high Infant and child mortality rates, low access to and utilization of family planning services and Inadequate healthcare systems compared to the developed worlds [22]. Importantly, Kenya experienced significant interregional disparities in fertility from a high of 7.8 births per woman in Wajir, situated in the North-Eastern part of Kenya, to a low of 2.3 births per woman in Kirinyaga counties in the central part of the country [22]. Kenya is among the three African countries that are considered as the pioneers of the transition within the SSA region with the first transition observed in 1980s [23]. Despite Kenya being a pioneer in transition, to the best of our knowledge, none of the previous studies on fertility in Kenyan had examined the determinants of children ever born in Kenya using the available national representatives demographic and health survey data especially among married women. Although childbearing outside of marital unions has increased in the recent past, it is the case that that most of the childbearing still takes place within marital unions in most African societies [15, 23], and the rate of marriage and marital fertility can have substantial impact on population-level patterns and childbearing [24] .The study bridges the gap by determining the factors associated with the children ever born and change in fertility among married women of reproductive age by decomposing fertility change between 2003 and 2014 in Kenya.

## Methods

### Data source

The study used datasets from the Kenya Demographic and Health Surveys (KDHS) of 2003 [25], 2008 [26], and 2014 [22]. The KDHS is a nationally representative cross-sectional survey that uses a two-stage sampling design: the selection of enumeration areas and households using a master household listing from each of the selected enumeration areas. All eligible women of reproductive age in the selected households who voluntarily consented to participate were interviewed by trained interviewers. The protocols for the surveys were reviewed and approved by a national ethics review board [22, 25, 26].

This study included all women aged 15–49 years that were married or in a union. Women who were not in a union (n = 20,836), those who did not respond to the question on fertility preference (n = 10,311), and those with missing age or aged below 10 years at their first sexual encounter (n = 1042), were excluded from the analysis.

**Table 1:** Respondents’ characteristics, 2003–2014

Characteristics		Year of survey												
		2003 n (%)		2008 n (%)		2014 n (%)		Period 2008–2003 (Percentage point diff)			Period 2014–2008 Percentage point diff)	Full period 2014–2003 Percentage point diff)		
Number of women (weighted)	3,917		4,002		7,332									
**Age groups**														
15–19		225 (5.7)		183 (4.6)		250 (3.4)		-1.1		-1.2		-2.3		
20–29		1627 (41.5)		1729 (43.2)		3085 (42.1)		1.7		-1.1		0.6		
30–39		1266 (32.3)		1325 (33.1)		2576 (35.1)		0.8		2.0		2.8		
40–49		799 (20.4)		765 (19.1)		1421 (19.4)		-1.3		0.3		-1.0		
**Education levels**														
None		523 (13.4)		404 (10.1)		634 (8.6)		-3.3		-1.5		-4.8		
Primary		2328 (59.4)		2341 (58.5)		3966 (54.1)		-0.9		-4.4		-5.3		
Secondary		830 (21.2)		975 (24.4)		2003 (27.3)		3.0		2.9		6.1		
Higher		235 (6.0)		282 (7.1)		729 (9.9)		1.1		2.8		3.9		
**Place of residence**														
Rural		3053 (77.9)		3054 (76.3)		4430 (60.4)		-1.6		-15.9		-17.5		
**Religion**														
Roman catholic		968 (24.8)		845 (21.1)		1402 (19.1)		-3.7		-2.0		-5.7		
Protestants/other Christian		2581 (66.0)		2752 (68.8)		5288 (72.1)		2.8		3.3		6.1		
Muslim		288 (7.4)		269 (6.7)		471 (6.4)		-0.7		-0.3		-1.0		
No religion/other religions		79 (2.0)		136 (3.4)		171 (2.3)		1.4		-1.1		0.3		
**Region**														
Nairobi		344 (8.8)		307 (7.7)		760 (10.4)		-1.1		2.7		1.9		
Central		558 (14.2)		427 (10.7)		930 (12.7)		-3.5		2.0		-1.5		
Coast		263 (6.7)		359 (9.0)		703 (9.6)		2.3		0.6		2.9		
Eastern		691 (17.6)		709 (17.7)		1181 (16.1)		0.1		-1.6		-1.5		
Nyanza		582 (14.9)		634 (15.9)		1083 (25.3)		1.0		9.4		10.4		
Rift valley		868 (22.2)		1040 (26.0)		1858 (25.3)		3.8		-0.7		3.1		
Western		503 (12.9)		471 (11.8)		644 (8.8)		-1.1		-3.0		-4.1		
North Eastern		108 (2.8)		55 (1.4)		173 (2.4)		-1.4		1.0		-0.4		
**Wealth Index**														
Lowest		728 (18.6)		676 (16.9)		1224 (16.7)		-1.7		-0.2		-1.9		
Second		772 (19.7)		737 (18.4)		1286 (17.5)		-1.3		-0.9		-2.2		
Middle		712 (18.2)		780 (19.5)		1388 (18.9)		1.3		-0.6		0.7		
Fourth		796 (20.3)		827 (20.7)		1581 (21.6)		0.4		0.9		1.3		
Highest		908 (23.2)		982 (24.5)		1852 (25.3)		1.3		0.8		2.1		
**Age at first sexual intercourse**														
Below 15		750 (19.1)		574 (14.3)		1198 (16.3)		-4.8		2.0		-2.8		
15–19		2497 (63.7)		2645 (66.1)		4638 (63.3)		2.4		-2.8		-0.4		
20+		670 (17.1)		784 (19.6)		1496 (20.4)		2.5		0.8		3.3		
**Age at first marriage**														
Below 18		1447 (37.0)		1355 (33.9)		2356 (32.1)		-3.1		-1.8		-4.9		
18–24		2175 (55.5)		2313 (57.8)		4267 (58.2)		2.3		0.4		2.7		
25+		294 (7.5)		330 (8.3)		709 (9.7)		0.8		1.4		2.2		
**Family size preferences**														
0–2		724 (18.5)		859 (21.5)		1676 (22.9)		3.0		1.0		4.4		
3–4		2075 (53.0)		2135 (53.4)		4020 (54.8)		0.4		1.4		1.8		
5+		1118 (28.5)		1007 (25.2)		1636 (22.3)		-3.3		-2.9		-6.2		
**Child mortality experience**														
One or more child deaths		1018 (26.0)		850 (21.2)		1275 (17.4)		-4.8		-3.8		-8.6		
**current use of contraceptive**														
Yes		2605 (66.5)		2989 (74.7)		5864 (80.0)		8.2		5.3		13.5		

n = weighted frequency (weighted per cent in the parenthesis); % diff: percentage difference

### Study variables

In this study main outcome of interest was measured as a discrete count of the number of children ever born alive (CEB) among married a woman of reproductive age (15–49 years) as at the time of the survey data collection (an expression of lifetime fertility). The independent variables included: age of the woman (15–19, 20–29, 30–39 and 40–49 years); level of education (no education, primary, secondary and higher); place of residence (rural or urban); the age of the woman a first sexual experience (< 15, 15–19 and 20–49 years); the age of the woman at first marriage (< 18, 18–24 and ≥ 25 years); family size preferences (0–2, 3–4, and ≥ 5); having experienced child mortality (Yes or No); religious affiliations (roman Catholics, protestant/other Christians, Muslims and no religion/other religious affiliations);region of residence (Nairobi, Central, Coast, Eastern, Nyanza, Rift Valley, Western, and North Eastern); current use of any contraceptive method (Yes or No); and household wealth Index(lowest, second, middle, fourth, highest). The household wealth Index is a standardized demographic and health survey measures of household economic status which is calculated by considering the assets owned by the households and the living conditions. These are weighted using the principal component analysis and the resulting index divided into five quintiles. Each household is classified to belong to one of these categories [27]. The inclusion of the above variables were based on previous studies [28–30] on the decomposition of these factors on fertility change.

### Data analysis

First, 2003, 2008 and 2014 datasets were merged and weighted using predefined women population-adjusted sampling weight to make sample data representative of the entire Kenyan population and account for the complex survey design. Second, frequency distributions were used to describe the sample characteristics for each survey year. The mean CEB was calculated for each survey and across sample characteristics while the significant difference in different time points was assessed using analysis of variance (ANOVA). The trend analysis for the characteristics and mean number of children was assessed phases for the periods phase I: 2003–2008, phase II: 2008–2014, and phase III: 2003–2014. Poisson regression model with robust standard errors was used to assess the relation between CEB and explanatory variables for each round of survey years. The association of explanatory variables to CEB was reported using an incidence rate ratio (95% confidence interval). Finally, a Poisson-based multivariate decomposition model for nonlinear responses (*mvdcmp* in Stata version 15) [31] was performed to identify factors contributing to the change in the mean number of children born between 2003 and 2014 with the aim of identifying the sources of changes in the CEB among married women during the period. The overall change over time in the mean number of children ever born was decomposed into two components: one that explains changes due to differences in observable characteristics (endowments) across the surveys and one that explains changes due to the different coefficients or effects (behaviour) over the study period. A p-value < 0.05 was set as the level of significance to determine statistical associations.

## Results

### Sample characteristics

The study included a weighted sample of 15,251 married women (3,917 in 2003, 4,002 in 2008, and 7,332 in 2014) (Fig. 1). Between 2003 and 2014, the proportion of women with secondary or higher levels of education increased from 27.2 to 37.2%. Similarly, the proportion of married women who were on contraceptives at the time of the survey significantly increased from 66.5 to 80.0% while those who had been married early (below 18 years) or experienced a child death decreased from 37.0 to 32.1% and from 26.0 to 17.4%, respectively (Table 1).

### Trends in cumulative fertility

**Fig. 2:**
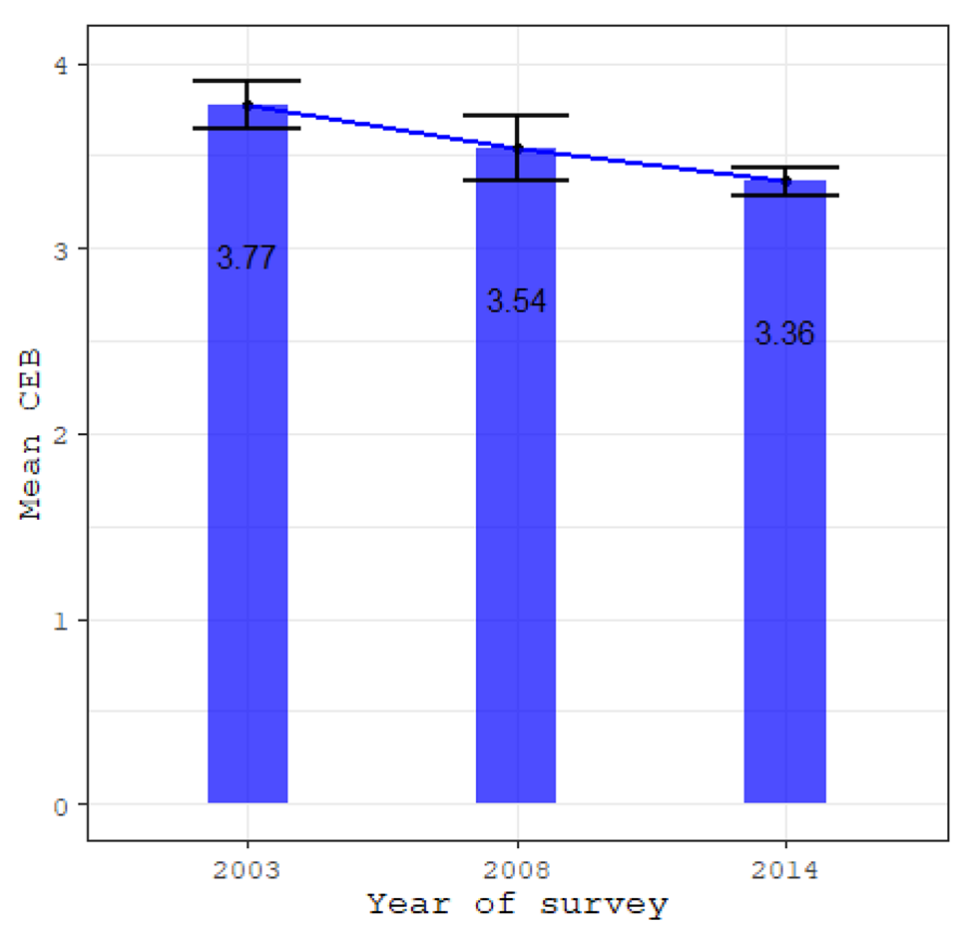
Trends in the mean number of children ever born, 2003 to 2014

Overall, the mean number of children ever born among married women significantly declined by 10.9% from 3.77 (95% CI: 3.64–3.90) in 2003 to 3.54 (95% CI: 3.37–3.72) in 2008 and 3.36 (95% CI: 3.28–3.44) in 2014 [p < 0.001] (Fig. 2). The mean number of children ever born varied significantly by level of education, wealth index, place of residence, family size preference, child mortality experience, and current use of contraceptives (Table 2).

**Table 2:** Trends in the mean number of children ever born by respondents’ characteristics

Characteristics	Year of survey			Mean difference			
	Mean (SD)	Mean (SD)	Mean (SD)	2003–2008	2008–2014	2003–2014	ANOVA
	2003	2008	2014				p-value
**Age group, years**							
15–19	0.85 (0.06)	0.80 (0.06)	0.89 (0.06)	-0.05	0.09	0.04	0.787
20–29	2.37 (0.05)	2.32 (0.06)	2.13 (0.04)	-0.05	-0.19	-0.24	0.400
30–39	4.57 (0.09)	4.20 (0.09)	3.97 (0.06)	-0.37	-0.23	-0.6	0.001
40–49	6.19 (0.13)	5.83 (0.18)	5.35 (0.09)	-0.36	-0.48	-0.84	< 0.001
**Level of education**							
None	5.23 (0.16)	5.00 (0.19)	4.80 (0.12)	-0.23	-0.2	-0.43	0.003
Primary	3.82 (0.07)	3.74 (0.08)	3.77 (0.05)	-0.08	0.03	-0.05	0.010
Secondary	3.03 (0.09)	2.83 (0.12)	2.59 (0.06)	-0.2	-0.24	-0.44	0.006
Higher	2.70 (0.13)	2.31 (0.13)	1.94 (0.07)	-0.39	-0.37	-0.76	< 0.001
**Wealth Index**							
Lowest	4.86 (0.13)	4.48 (0.12)	4.44 (0.08)	-0.38	-0.04	-0.42	< 0.001
Second	4.25 (0.12)	4.23 (0.12)	3.94 (0.08)	-0.02	-0.29	-0.31	0.012
Middle	4.08 (0.12)	3.95 (0.15)	3.85 (0.09)	0.45	-0.1	-0.23	0.001
Fourth	3.50 (0.10)	3.31 (0.11)	2.87 (0.07)	-0.19	-0.44	-0.63	< 0.001
Highest	2.48 (0.07)	2.26 (0.08)	2.29 (0.06)	-0.22	0.03	-0.19	0.002
**Place of residence**							
Urban	2.61 (0.08)	2.27 (0.08)	2.52 (0.05)	-0.34	0.25	-0.09	< 0.001
Rural	4.10 (0.07)	3.94 (0.08)	3.91 (0.05)	-0.16	-0.03	-0.19	0.031
**Religion**							
Roman catholic	3.80 (0.12)	3.58 (0.12)	3.26 (0.08)	-0.22	-0.32	-0.54	0.236
Protestants/other Christian	3.75 (0.07)	3.51 (0.10)	3.29 (0.05)	-0.24	-0.22	-0.49	0.011
Muslim	3.97 (0.18)	3.47 (0.14)	4.04 (0.11)	-0.50	0.57	0.07	< 0.001
No religion/other religions	3.53 (0.31)	4.14 (0.29)	4.48 (0.29)	0.61	0.34	0.95	0.003
**Region**							
Nairobi	2.29 (0.09)	2.07 (0.08)	2.21 (0.14)	-0.22	0.14	-0.08	0.067
Central	3.15 (0.11)	3.07 (0.13)	2.79 (0.08)	-0.08	-0.28	-0.36	0.033
Coast	3.33 (0.17)	3.32 (0.19)	3.46 (0.12)	-0.01	0.14	0.13	0.105
Eastern	3.72 (0.17)	3.71 (0.16)	3.30 (0.10)	-0.01	-0.41	-0.42	0.009
Nyanza	4.59 (0.18)	3.48 (0.12)	3.77 (0.09)	-1.11	0.29	-0.82	< 0.001
Rift valley	3.89 (0.14)	3.82 (0.24)	3.50 (0.08)	-0.07	-0.32	-0.39	0.001
Western	4.33 (0.13)	4.25 (0.23)	4.10 (0.15)	-0.08	-0.15	-0.23	0.482
North Eastern	5.11 (0.28)	4.36 (0.20)	4.60 (0.12)	-0.75	0.24	-0.51	0.028
**Age at first sexual intercourse**							
Below 15	4.62 (0.14)	4.62 (0.14)	4.22 (0.08)	0	-0.4	-0.4	0.072
15–19	3.72 (0.07)	3.54 (0.09)	3.43 (0.05)	-0.18	-0.11	-0.29	0.006
20+	3.00 (0.11)	2.76 (0.10)	2.44 (0.07)	-0.24	-0.32	-0.56	0.012
**Age at first marriage**							
Below 18	4.48 (0.09)	4.40 (0.11)	4.19 (0.06)	-0.08	-0.21	-0.29	0.251
18–24	3.41 (0.07)	3.14 (0.09)	3.06 (0.05)	-0.27	-0.08	-0.35	0.001
25+	2.96 (0.14)	2.87 (0.15)	2.38 (0.10)	-0.09	-0.49	-0.58	0.041
**Family size preferences**							
0–2	2.57 (0.09)	2.48 (0.11)	2.51 (0.06)	-0.09	0.03	-0.06	0.024
3–4	3.62 (0.70)	3.33 (0.08)	3.09 (0.04)	-0.29	-0.24	-0.53	< 0.001
5+	4.83 (0.09)	4.91 (0.13)	4.88 (0.08)	0.08	-0.03	0.05	0.416
**Child mortality experience**							
No child death	3.02 (0.05)	3.04 (0.80)	2.91 (0.04)	0.02	-0.13	-0.11	< 0.001
≥ 1 deaths	5.92 (0.09)	5.42 (0.12)	5.50 (0.09)	-0.5	0.08	-0.42	< 0.001
**Ever used contraceptive**							
No	3.82 (0.11)	3.55 (0.12)	3.54 (0.10)	-0.27	-0.01	-0.28	0.012
Yes	3.74 (0.06)	3.54 (0.10)	3.31 (0.04)	-0.2	-0.23	-0.43	0.002
**Total**	**3.77 (0.08)**	**3.54 (0.09)**	**3.36 (0.04)**	**-0.23**	**-0.18**	**-0.41**	**< 0.001**

SD: Standard deviation

### Determinants of lifetime fertility: Poisson regression model

The results of the regression models for the determinants of CEB for different survey rounds are shown in Table 3. Across the survey years, there was no significant association between CEB and being in 30–39 years age-group, having a primary level of education, being a resident of coast region and being affiliated with protestant/other Christian or no religion. The expected number of children reduced with the age at first sexual Intercourse and the age at first marriage across the survey years. on the other hand, the expected number of children increased with the increase with the number of family size preferences. Women who had lost one or more children in the past were likely to have increased number of children. women from Rift-valley, Western and North eastern had significantly increase in CEB across the years as compared with women from Nairobi area. Finally, the number of children decreased with the increase in the household wealth Index. Women from households with highest wealth index were likely to have decreased number of children as compared to their counterparts in the households with lowest wealth Index.

**Table 3:** The incidence rate ratios of CEB and explanatory variables among women of reproductive age in Kenya 2003–2014

Characteristics	Year of survey		
	AIRR (95%CI)	AIRR (95%CI)	AIRR (95%CI)
	2003	2008	2014
**Age-group**			
15–19	0.32*** (0.29–0.36)	0.35*** (0.32–0.40)	0.34*** (0.31–0.38)
20–29	0.73*** (0.70–0.75)	0.77*** (0.75–0.80)	0.74*** (0.72–0.76)
30–39	0.99 (0.96–1.02)	1.03 (0.99–1.06)	0.99 (0.97–1.01)
40–49	Reference	Reference	Reference
**Level of education**			
None	Reference	Reference	Reference
Primary	1.05 (0.99–1.10)	0.99 (0.95–1.05)	0.97 (0.94–1.03)
Secondary	0.96 (0.91–1.02)	0.93 **(0.88–0.99)	0.88*** (0.85–0.92)
Higher	0.93 (0.86–1.01)	0.84*** (0.78–0.91)	0.78*** (0.74–0.82)
**Place of residence**			
Urban	0.96 (0.91-1.00)	0.96*** (0.91–1.01)	0.96*** (0.94–0.98)
Rural	Reference	Reference	Reference
**Age at first sexual intercourse**			
Below 15	Reference	Reference	Reference
15–19	0.94** (0.91–0.98)	0.89*** (0.86–0.92)	0.93** (0.91–0.95)
20+	0.84*** (0.79–0.88)	0.77*** (0.73–0.81)	0.80*** (0.78–0.83)
**Age at first marriage**			
Below 18	Reference	Reference	Reference
18–24	0.87*** (0.84–0.89)	0.86*** (0.83–0.86)	0.86*** (0.85–0.88)
25+	0.71*** (0.66–0.76)	0.69*** (0.65–0.74)	0.68*** (0.65–0.71)
**Family size preferences**			
0–2	Reference	Reference	Reference
3–4	1.10*** (1.06–1.14)	1.10*** (1.06–1.14)	1.07*** (1.04–1.10)
5+	1.19*** (1.14–1.24)	1.25*** (1.20–1.30)	1.20*** (1.16–1.24)
**Child mortality experience**			
No child death	Reference	Reference	Reference
One or more deaths	1.33*** (1.29–1.37)	1.25*** (1.21–1.29)	1.28*** (1.25–1.31)
**Wealth Index**			
Lowest	Reference	Reference	Reference
Second	0.95*** (0.91–0.98)	0.99 (0.95–1.03)	0.97*** (0.94–0.99)
Middle	0.94** (0.90–0.98)	0.91*** (0.87–0.95)	0.92** (0.89–0.95)
Fourth	0.87** (0.83–0.91)	0.86*** (0.82–0.90)	0.83*** (0.81–0.86)
Highest	0.79*** (0.74–0.84)	0.76*** (0.71–0.81)	0.77*** (0.74–0.80)
**Regions**			
Nairobi	Reference	Reference	Reference
Central	1.02 (0.95–1.09)	0.96 (0.89–1.04)	0.90** (0.84–0.97)
Coast	1.01 (0.93–1.09)	1.06 (0.99–1.13)	0.97 (0.90–1.05)
Eastern	1.08** (1.04–1.16)	1.04 (0.97–1.12)	0.99 (0.91–1.06)
Nyanza	1.10*** (1.03–1.18)	1.07 (0.99–1.16)	1.06 (0.98–1.15)
Rift valley	1.18*** (1.10–1.27)	1.16*** (1.09–1.25)	1.09** (1.01–1.17)
Western	1.14*** (1.07–1.23)	1.10** (1.03–1.18)	1.11** (1.03–1.21)
North Eastern	1.28*** (1.14–1.43)	1.21*** (1.10–1.34)	1.11** (1.02–1.22)
**Religion**			
Roman catholic	Reference	Reference	Reference
Protestants/other Christian	1.01 (0.98–1.04)	0.99 (0.96–1.03)	0.99 (0.98–1.02)
Muslim	1.03 (0.95–1.12)	0.95 (0.90–1.01)	1.07*** (1.02–1.12)
No religion/other religions	0.99 (0.90–1.08)	0.96 (0.89–1.03)	1.01 (0.96–1.07)

***p < 0.001;**p < 0.05; AIRR, adjusted Incidence rate ratios

### Decomposition analysis

The overall decline in the mean number of children ever born between 2003 and 2014 was mainly due to differences in the coefficients (effects of characteristics) across the surveys, which explained 96.42% and the remaining percentage explained by the change in characteristics (endowment) (Table 3). All the characteristics (endowment) of the women showed no significant associated with the difference in lifetime fertility (Table 4). After controlling for the effects of other variables in the decomposition analysis, the difference in lifetime fertility between 2003 and 2014 was associated with the differences in women’s level of education. Thus, a decrease in the proportion of women’s attainment of primary education over the survey period (Table 1) showed a significant association to the differences in the lifetime fertility (Table 5). An increase in the proportion of women’s attaining secondary and higher level of education showed a significant positive association in the lifetime fertility by 35.10% and 26.52% respectively (Table 5).

**Table 4:** Determinants of the change in the mean number of CEB from 2003 to 2014

Component	Coefficient	P value	95%CI	%
E	0.208	0.782	-1.268–1.684	3.58
C	5.599	< 0.001	2.824–8.373	96.42
R	5.807	< 0.001	3.455–8.158	

**Table 5:** Decomposition analysis of change in cumulative fertility in Kenya between 2003 and 2014

	Endowments			Coefficients		
Variables	Coef	95%CI	pct	Coef	95%CI	pct
Age -group, years						
15–19	0.178	-1.170–1.526	3.06	-0.224	-0.997–0.549	-3.85
20–29	0.013	-0.083–0.108	0.22	-0.856	-3.323–1.611	-14.74
30–39	-0.001	-0.012–0.011	-0.02	-0.162	-1.904–1.579	-2.80
40–49	Ref	Ref	Ref	Ref	Ref	Ref
**Level of education**						
None	Ref	Ref	Ref	Ref	Ref	Ref
Primary	-0.004	-0.034–0.026	-0.07	3.978***	0.182–7.774	68.51
Secondary	-0.002	-0.021–0.016	-0.04	2.038***	0.441–4.081	35.10
Higher	-0.007	-0.066–0.052	-0.13	1.539***	0.429–2.650	26.52
**Place of residence**						
Urban	-0.022	-0.211–0.167	-0.38	-0.03	-2.466–2.406	-0.52
Rural	Ref	Ref	Ref	Ref	Ref	Ref
**Age at first sexual intercourse,years**						
Below 15	Ref	Ref	Ref	Ref	Ref	Ref
15–19	0.003	-0.018–0.023	0.05	1.168	-2.276–4.611	20.11
20+	-0.006	-0.055–0.042	-0.11	0.774	-0.842–2.389	13.32
**Age at first marriage,years**						
Below 18	Ref	Ref	Ref	Ref	Ref	Ref
18–24	-0.003	-0.026–0.021	-0.05	0.414	-2.354–3.182	7.13
25+	-0.02	-0.168–0.129	-0.34	0.395	-0.448–1.239	6.81
**Family size preference**						
0–2	Ref	Ref	Ref	Ref	Ref	Ref
3–4	0.002	-0.011–0.014	0.03	1.309	-1.940–4.558	22.54
5+	-0.021	-0.179–0.138	-0.36	-0.251	-2.427–1.925	-4.32
**Child mortality experience**						
No child death	Ref	Ref	Ref	Ref	Ref	Ref
One or more deaths	-0.16	-1384–1.064	-2.76	0.669	-0.187–1.526	11.53
**Wealth Index**						
Lowest	Ref	Ref	Ref	Ref	Ref	Ref
Second	-0.006	-0.053–0.041	-0.11	-0.513	-1.764–0.738	-8.83
Middle	-0.008	-0.067–0.051	-0.13	0.42	-0.859–1.700	7.24
Fourth	-0.004	-0.034–0.026	-0.07	0.836	-0.569–2.242	14.4
Highest	0.161	-1.036–1.358	2.77	0.451	-1.327–2.228	7.76
**Regions**						
Nairobi	Ref	Ref	Ref	Ref	Ref	Ref
Central	-0.007	-0.065–0.051	-0.12	1.147	-0.229–2.523	19.75
Coast	0.001	-0.016–0.019	0.02	0.466	-1.231–2.163	8.02
Eastern	0.028	-0.174–0.231	0.49	1.678	-0.885–4.240	28.89
Nyanza	0.018	-0.113–0.148	0.31	0.584	-1.505–2.674	10.06
Rift valley	0.159	-1.008–1.326	2.74	2.369	-1.596–6.333	40.8
Western	-0.057	-0.483–0.369	-0.98	0.217	-0.915–1.350	3.74
North Eastern	-0.027	-0.232 -0 0.178	-0.47	0.832	-0.204–1.868	14.34
**Religion**						
Roman catholic	Ref	Ref	Ref	Ref	Ref	Ref
Protestants/other Christian	-0.004	-0.046–0.039	-0.06	0.512	-2.222–3.247	8.82
Muslim	0.006	-0.047–0.059	0.10	2.241	-6.898-11.380	38.59
No religion/other religions	-0.003	-0.002–0.001	-0.01	-0.166	-2.299–1.967	-2.86

Ref, reference category; pct, percent; Coef, coefficient; ***significant at p < 0.001; CI, confidence Interval

## Discussion

Lifetime fertility for the developing countries remains a problem of public health concern and is Influenced by several sociodemographic and socioeconomic related factors. Between 2003 and 2014, the lifetime fertility in Kenya declined from a mean of 3.77 children ever born to 3.36; an 11% reduction, which was majorly explained by the differences in the effects of socio-demographic and socioeconomic characteristics of women between the survey years. Specifically, only level of education contributed significantly to the decline.

Over the study period, Kenya achieved substantial improvement in socioeconomic development. The proportion of women living in urban settings, those who had basic primary and secondary education and those who used contraceptives increased while the rate of early marriages and child mortality reduced. There was also increased sensitization and investment to promote family planning, birth spacing, and manageable families [32, 33], including committing to the FP2020 goals [34].

Girls’ education is associated with a marked decline in fertility in sub-Saharan Africa, including Kenya [35]. In 2003, Kenya implemented universal free primary education resulting in a higher enrolment of boys and girls in schools, even among poor communities [36]. The increased number of girls in school possibly delayed their sexual debut and marriage. In addition, in 2008, the country began implementing subsidised secondary education, allowing primary school pupils to easily transition to secondary schools while also lowering the entry requirements for girls to colleges and universities. Further to these new policies and increased investment in education, other interventions such as the campaigns against early marriages and female genital mutilation and access to sex education were implemented through the Children’s Act 2001 [37]. These interventions may partly explain the one-fifth contribution of the increase in the proportion of women with a secondary or higher level of education during the study period to the reduction in fertility as well as the reduced proportion of married teenagers. Higher education for women reduces the opportunity of getting married early and having large families, leading women to have fewer children [38]. Increasing levels of education operate on different pathways to reduce fertility, including its association with reductions in infant and child mortality, increased participation of women in the labour market, and increased use of contraception [39, 40]. Higher age at first marriage had a significant positive effect on women’s social and economic progression and reduced their fertility period because it excluded the teenage years. Delayed marriage, reflective of increased levels of education among women, also reduces the need for more children and the years available for childbearing making difficult for a woman to conceive, thus influencing fertility [41].

This study also found that the number of children Increased among women who had experienced in child mortality across the survey years; a finding observed in other settings [42, 43]. During that period, the infant mortality and under-5 mortality rates declined from 53.1 to 36.5 deaths per 1000 live births and from 85.5 to 43.2 deaths per 1000 live births, respectively [44]. Infant mortality affects fertility through replacement effects and biological changes. Previously, women would give birth to many children as a replacement strategy for children at risk of death due to various causes. Improved maternal healthcare services, better economic status and livelihood have contributed to reduced deaths hence reducing the need for more children. Biological effects can be explained by the interruption of breastfeeding in case of infant death, which shortens the postpartum amenorrhea period as the mother can conceive sooner, leading to shorter birth intervals and higher fertility [42].

Urban residence was associated with low number of children in reference to the rural areas. During the study period, the proportion of married women living in urban areas increased by 17.5%, contributing to approximately half of the decrease in lifetime fertility. Living in urban areas increases the direct cost of childbirth and opportunity costs due to the numerous avenues of socioeconomic mobility associated with increased industrial employment and higher education infrastructure [45]. Urban residents also tend to have better access to all types of services and information, an improved level of education, more employment opportunities and a preference for smaller families in urban areas [45].

Early exposure to sexual intercourse increases the probability of early pregnancy and childbirth, since, at an early age, there is limited awareness and use of condoms and other contraceptives resulting in unplanned pregnancies [46]. Women who had their first sexual intercourse as teenagers had a negative effect on fertility changes between 2003 and 2014. The finding is consistent with studies in Uganda among women who have ever had sex [47]. Women who delayed sexual intercourse to age 20 and over showed a positive contribution to the change in fertility. Delayed sexual intercourse shortens the childbearing period and implies delayed exposure to pregnancy and childbearing.

Similarly, the study showed that women from households with highest wealth index had lower number of children compared to those from the lowest wealth index. This was consistent with a study In Ethiopia and Kenya [48] where women in the fourth and highest indices had reduced number of children. Women from these households are likely to access to different family planning methods and information on the benefit of the same. They are also likely to be employed and would not like childbearing to be constantly interfering with their employment activities, thus prefers a smaller family size.

### Strength and limitations

This is the first study in Kenya on factors determining the CEB and its change conducted in Kenya using the decomposition analysis technique from existing KDHS datasets. The study used public nationally representative data with high response rates to assess the general trends and quantified the contribution of factors to the change in cumulative fertility. The decomposition of the factors that drive fertility change provided a rich analysis set for policy interventions to address socioeconomic factors influencing fertility change in Kenya. The study used datasets collected up to 2014, and circumstances could have changed since then hence a need for studies to update these findings when the most recent data is available to provide additional evidence on the current fertility trajectory in Kenya and the contribution of these factors to driving fertility change. The study did not include some of the factors such as ethnicity, culture, government policies that could better provide explanations to the changes observed in CEB to perform the analysis because they were not available. The current study may be used as the basis for future qualitative studies that include some of these factors that Influence CEB. The study used data from three cross-sectional surveys that makes It difficult to establish causal relationship between CEB and explanatory variables since the outcome and explanatory measures are determine at the same time, therefore further study on causality is essential.

## Conclusion

Kenya experienced a decrease in cumulative fertility between 2003 and 2014 despite a temporary stall between 2003 and 2008. The expected number of children reduced with the age at first sexual intercourse, the age at first marriage, and household wealth Index across the survey years. However, the expected number of children increased with the increase with the number of family size preferences, death 0f one or more children in the past, in Rift-valley, Western and North eastern regions. Changes in the effects of women’s socio-demographic and socioeconomic characteristics during this period explained much of the decline specifically the effect of women’s level of education. We call for continued strengthening of current interventions and policies on population and development in Kenya including increased prioritisation and investment in higher education for boys and girls, sex behaviour change communication, and awareness especially in rural settings. In addition, other maternal, newborn and child health interventions geared towards the reduction of child mortality should be further strengthened for Kenya to attain its population and sustainable development goals by 2030. The findings offer appropriate policy directions for fertility regulation programs in the country that can help in improving maternal and newborn morbidity and mortality.

## Data Availability

Demographic and health survey (DHS) datasets are available freely from the DHS website (https://dhsprogram.com/data/available-datasets.cfm). The datasets are available after registration on the website.
